# The diversity among the species *Tetragenococcus halophilus* including new isolates from a lupine seed fermentation

**DOI:** 10.1186/s12866-021-02381-1

**Published:** 2021-11-20

**Authors:** Tobias Link, Rudi F. Vogel, Matthias A. Ehrmann

**Affiliations:** Lehrstuhl für Mikrobiologie, Technische Universität München, 85354 Freising, Germany

**Keywords:** Lupine moromi, *Tetragenococcus halophilus*, Starter culture, Phylogenetic lineages

## Abstract

**Background:**

*Tetragenococcus (T.) halophilus* can be isolated from a variety of fermented foods, such as soy sauce, different soy pastes, salted fish sauce and from cheese brine or degraded sugar beet thick juice. This species contributes by the formation of short chain acids to the flavor of the product. Recently, *T. halophilus* has been identified as a dominant species in a seasoning sauce fermentation based on koji made with lupine seeds.

**Results:**

In this study we characterized six strains of *T. halophilus* isolated from lupine moromi fermentations in terms of their adaptation towards this fermentation environment, salt tolerance and production of biogenic amines. Phylogenic and genomic analysis revealed three distinctive lineages within the species *T. halophilus* with no relation to their isolation source, besides the lineage of *T. halophilus* subsp. *flandriensis*. All isolated strains from lupine moromi belong to one lineage in that any of the type strains are absent. The strains form lupine moromi could not convincingly be assigned to one of the current subspecies. Taken together with strain specific differences in the carbohydrate metabolism (arabinose, mannitol, melibiose, gluconate, galactonate) and amino acid degradation pathways such as arginine deiminase pathway (ADI) and the agmatine deiminase pathway (AgDI) the biodiversity in the species of *T. halophilus* is greater than expected. Among the new strains, some strains have a favorable combination of traits wanted in a starter culture.

**Conclusions:**

Our study characterized *T. halophilus* strains that were isolated from lupine fermentation. The lupine moromi environment appears to select strains with specific traits as all of the strains are phylogenetically closely related, which potentially can be used as a starter culture for lupine moromi. We also found that the strains can be clearly distinguished phylogenetically and phenotypically from the type strains of both subspecies *T. halophilus* subsp. *halophilus* and *T. halophilus* subsp. *flandriensis*.

**Supplementary Information:**

The online version contains supplementary material available at 10.1186/s12866-021-02381-1.

## Introduction

The genus *Tetragenococcus* (*T.*) currently comprises five different species including *T. osmophilus, T. muriaticus, T. solitarius, T. koreensis* and *T. halophilus* with the two subspecies *T. halophilus* subsp. *halophilus* and *T. halophilus* subsp. *flandriensis* [1–5]. Tetragenococci are Gram-positive, non-motile, facultative anaerobic lactic acid bacteria adapted to high sugar or high salinity habitats. *T. halophilus* is characterized by its high NaCl tolerance up to 20 %, growth at pH 7 - 9, but a moderate acid tolerance below pH 5 [6–8]. Thus, *T. halophilus* strains are typically isolated from fermented foods containing high amounts of NaCl such as soy sauce moromi, soy pastes or different variants of fermented fish products [9–11]. This species contributes to the fermentation by the production of organic acids as well as the degradation of unfavoured sugars [12–14]. Moreover, *T. halophilus* strains are able to prevent the growth of different autochthonous strains harboring the potential to produce biogenic amine (BA) and thereby reduce the BA content in the final product [15]. For some strains a degradation of aflatoxins is reported [16]. The ability of *T. halophilus* to survive in high saline environments is mainly due to the import of compatible solutes such as glycine betaine, proline and choline via the OpuA, OpuC, OpuD and BetT transporters as the intracellular accumulation of glycine betaine and proline is favored under high saline conditions [17–20]. Besides the import of these compatible solutes the ADI pathway is also upregulated under high saline conditions generating ammonia and increasing the intracellular citrulline content [21]. Under saline conditions the glutamate dehydrogenase as well as the Na^+^ translocating V-Type ATPase were also more abundant [22].

Although the majority of isolates come from high salt soy and fish fermentations, there are also more recent reports on isolates from degraded sugar beet thick juice, Brie de meaux cheese rind, mountain snow and from water samples [5, 23–25].

This study reports on the characterization of previously isolated *T. halophilus* strains occurring in a seasoning sauce fermentation based on koji made from lupine seeds [41]. We investigated these strains to see whether and to what extent the use of the hitherto uncommon substrate lupine selects for specifically adapted isolates and propose traits, which useful in the selection of starter cultures. To reveal genomic differences within the species we used a comparative genomics approach supported by physiological data. Furthermore, we characterized the new strains with regards to their salt tolerance, formation of biogenic amines, carbohydrate metabolism and bacteriocin production.

## Results

### Genomic diversity of *Tetragenococcus halophilus*

The genomes of six isolates from lupine moromi as well as from DSM 20337 were sequenced. The genome sequences of the isolates from lupine moromi were compared to previously published genomes available in a public database (NCBI). In order to avoid using sequences that are identical we set an ANIb (average nucleotide indices based on Blast) value cutoff of 99.6 % for the selection of strains from the NCBI database (Table 1). To further ensure that the set of strains used was representative for the species a pan/core plot was generated (Fig. S1). As the number of genes included in the core genome only slightly decreased after seventeen strains the core genome was considered as closed. The genome sizes of *T. halophilus* strains ranges from 2.26 to 2.6 Mb and the GC content ranges from 35.55 to 36.32 % (Table 1). Member of this species only sporadically carry plasmids as the type strain *T. halophilus* subsp. *flandriensis* DSM 23766^T^ is carrying one plasmid and the strains TMW 2.2256, TMW 2.2257 and TMW 2.2263 are predicted to carry a plasmid as it can be derived from the genomic sequences.

**Table 1:** Strains used, accession numbers and characteristics of their genomes

Accession number	Strain name	References	Origin	Genomsize (bp)	Contigs	Plasmids	G/C content (%)
CP027783 (1-3)	*T. osmophilus* DSM 23765^T^	Juste *et al.,* 2012	Degraded sugar thick juice	2329167	3	2	36.34
CP027768 (1-2)	*T. halophilus* subsp. *flandriensis* DSM 23766^T^	Juste *et al.,* 2012	Degraded sugar thick juice	2724800	2	1	36.32
NZ_PXYA01000000	*T. halophilus* subsp*. halophilus* DSM 20339^T^	Chun *et al.,* 2019	Salted anchovy	2595756	5		36.03
NC_016052	*T. halophilus* subsp*. halophilus* NBRC 12172	NITE 2011	Soy sauce mash	2562720	1		36.04
CP012047	*T. halophilus* MJ4	Kim *et al.,* 2019	Fish (anchovy) sauce	2389470	1		35.99
CP046246	*T. halophilus* YJ1	Yonsei University	Salty fish sauce	2476596	1		36.12
CP020017	*T. halophilus* KUD23	Lee *et al.,* 2018	Korean soypaste doenjang	2599117	1		36.06
NZ_LSFG00000000	*T. halophilus* FBL3	Kim *et al.,* 2017	Galchijeot, fish sauce	2420904	87		35.75
BLRM01000000	*T. halophilus* WJ7	Shirakawa *et al*., 2020	Fish nukazuke with rice bran	2443531	113		35.56
JACABZ000000000	*T. halophilus* TMW 2.2254	This study	Lupine moromi	2401046	77		35.7
JACACA000000000	*T. halophilus* TMW 2.2256	This study	Lupine moromi	2407620	101	1	35.73
JACACB000000000	*T. halophilus* TMW 2.2257	This study	Lupine moromi	2483454	92	1	35.69
JACACH000000000	*T. halophilus* TMW 2.2263	This study	Lupine moromi	2459525	98	1	35.74
JACACI000000000	*T. halophilu*s TMW 2.2264	This study	Lupine moromi	2308230	76		35.7
JACACK000000000	*T. halophilus* TMW 2.2266	This study	Lupine moromi	2388983	97		35.78
JACTUX000000000	*T. halophilus* subsp. *halophilus* DSM 20337	This study	Soy sauce mash	2259739	112		35.67
NZ_BDDZ00000000	*T. halophilus* subsp*. halophilus* 11	Nishimura *et al.,* 2017	Soy sauce mash	2410712	311		35.55
NZ_BDEH00000000	*T. halophilus* subsp. *halophilus* D10	Nishimura *et al.,* 2017	Soy sauce mash	2438535	259		35.63
NZ_BDEI00000000	*T. halophilus* subsp*. halophilus* D-86	Nishimura *et al.,* 2017	Soy sauce mash	2455970	315		35.61
NZ_BDEC00000000	*T. halophilus* subsp*. halophilus* NISL 7118	Nishimura *et al.,* 2017	Soy sauce mash	2401304	281		35.72
NZ_BDEF00000000	*T. halophilus* subsp. *halophilus* NISL 7126	Nishimura *et al.,* 2017	Soy sauce mash	2511812	214		35.71
JABEVO010000000	*T. halophilus* KG12		Korean soy sauce	2515188	117		35.69
BLRP01000000	*T. halophilus* YG2	Shirakawa *et al*., 2020	Soy sauce mash	2372244	117		35.67
BLRO01000000	*T. halophilus* YA5	Wakinaka *et al*., 2019	Soy sauce mash	2318625	122		35.72
BLRN01000000	*T. halophilus* YA163	Shirakawa *et al*., 2020	Soy sauce mash	2420090	132		35.7
BKBL01000000	*T. halophilus* 8C7	Unno *et al.,* 2020	Brie de meaux cheese rind	2344983	132		35.85

**Fig. 1:**
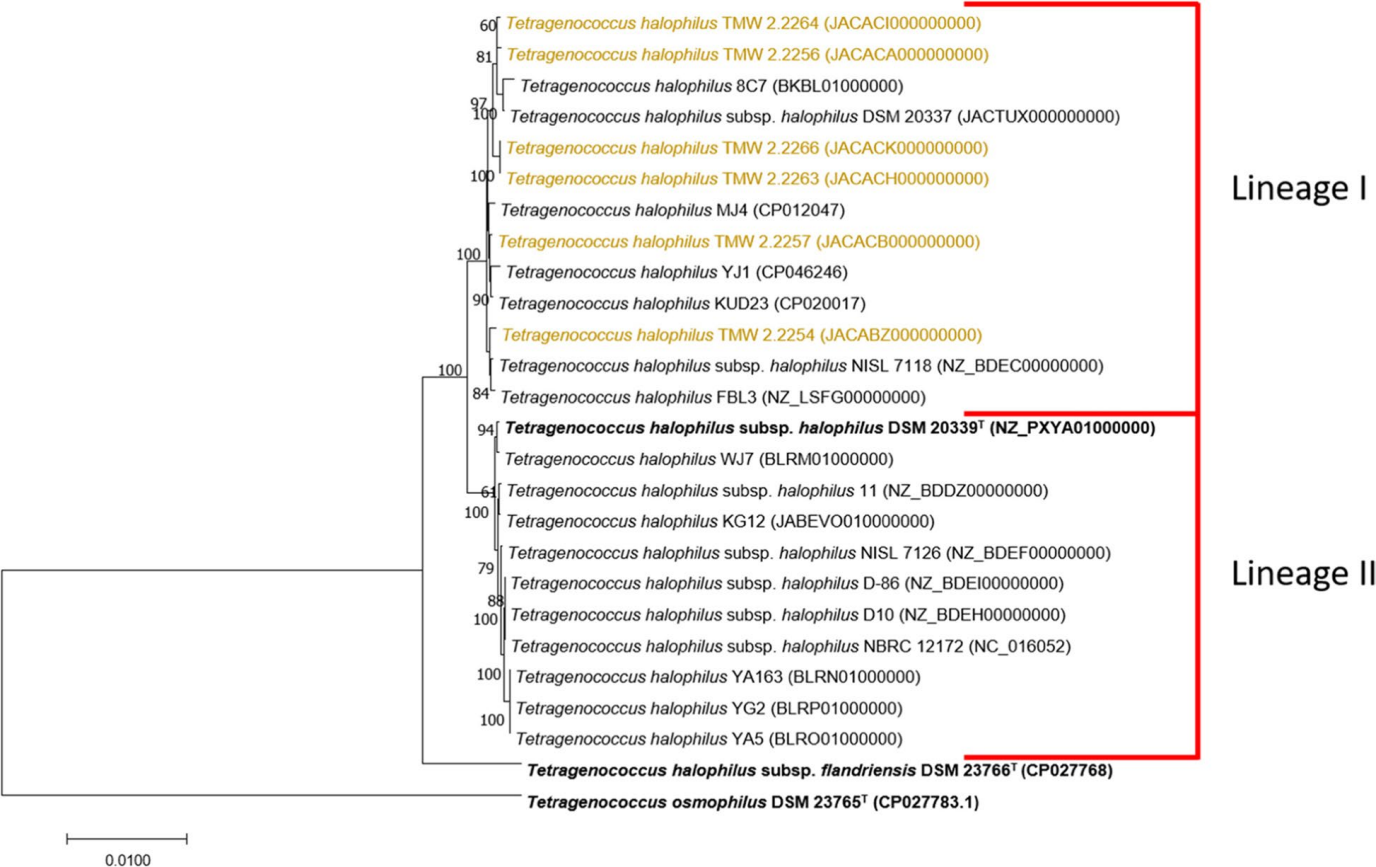
Phylogenetic tree of *T. halophilus* based on concatenated nucleotide sequences of the housekeeping genes (*fusA*, *gyrA*, *gyrB*, *lepA*, *pyrG*, *recA*, *rpoD*) using the Neighbor-Joining method [49]. The optimal tree with the sum of branch length = 0.10333351 is shown. The percentage of replicate trees in which the associated taxa clustered together in the bootstrap test (1000 replicates) are shown next to the branches [52]. The tree is drawn to scale, with branch lengths in the same units as those of the evolutionary distances used to infer the phylogenetic tree. The evolutionary distances were computed using the Maximum Composite Likelihood method [53] and are in the units of the number of base substitutions per site. All positions containing gaps and missing data were eliminated. There were a total of 12057 positions in the final dataset. The strains isolated from lupine moromi are marked in yellow.* T. osmophilus *DSM23765^T^ was used as an outgroup

Sequence similarity of 16 S rRNA genes and of concatenated housekeeping genes, the ANIb values and the *in-silico* DDH (DNA-DNA hybridization) values were calculated and used to define the genomic relationship of the strains to each other. The phylogenetic tree from the alignment of the 16 S rRNA gene revealed that all of the isolates from lupine moromi are belonging to the species *T. halophilus* (Fig. S2). To further analyze the phylogenetic relatedness, seven marker genes (*fusA*, *gyrA*, *gyrB*, *lepA*, *pyrG*, *recA*, *rpoD*) were used to construct phylogenetic trees (Fig. 1 and Fig. S3). This revealed three distinctive lineages within the *T. halophilus* species. The first includes 13 strains from varying origin, all strains isolated from lupine moromi were present within this lineage. The second lineage includes 11 strains and the type strain *T. halophilus* subsp. *halophilus* DSM 20339^T^. The third lineage consisted only of the type strain *T. halophilus* subsp. *flandriensis* DSM 23766^T^. This separation into these three lineages is also supported by the ANIb values. With the first lineage being the most divers one with a median ANIb value of 97.4. The second lineage is more alike with an intra-lineage median ANIb value of 98.4. The inter-lineage median ANIb value when comparing lineage I vs. lineage II is 96.8. These values support the separation of both lineages but still do not allow a separation into two subspecies. In order to further determine the subspecies affiliation of our isolates the genomic distance of the type strains *T. halophilus* subsp. *halophilus* DSM 20339^T^ and *T. halophilus* subsp. *flandriensis* DSM 23766^T^ was calculated using the GGDC 2.1 calculator. The *in-silico* DDH values of all of the new isolates from lupine moromi were <79 % when compared to the type strain DSM 20339^T^ or to DSM 23766^T^.

Comparative analysis for CDS only present in either lineage revealed no specific CDS for lineage I could be found that is present in all strains, but in eleven of thirteen strains a PTS IIC component, a LacI regulator and an alpha-L-fucosidase are present. Besides that, in another constellation of eleven strains from lineage I a fumarate reductase subunit, a hypothetical protein and a subunit *opuAC* are present, which are absent in strains from lineage II (Table S2).

As the total number specific CDS for lineage II is lower compared to lineage I, lineage II appears to be more homogenous. Ten CDS could be found that are unique to lineage II. Among these, the most interesting CDS is the hsp33 family molecular chaperone HslO. Although not present in all strains form lineage II, it is notable that a putative glycerol sn-3- phosphate transporter and a putative pentose ABC transporter *xylEFG* are present only in the strains from lineage II (Table S2).

### Analysis of carbohydrate metabolism

As utilization of available carbohydrates is essential for the growth and is the main way of *T. halophilus* for generation of ATP, the carbohydrate metabolisms can be quite diverse depending on the isolation source [6, 10, 26–29]. We therefore looked at the carbohydrate metabolism of each strain. Using BADGE variations in the abundance of the carbohydrate metabolizing cluster L-arabinose, D-ribose, D-xylose, D-galactose, D-lactose, D-melibiose, D-mannitol, D-sorbitol, gluconate and galactonate were found in most of the strains of the set used in this study. Functional metabolic cluster prediction based on the NCBI, RAST, TIGR and KEGG annotations were analyzed for the entire set and clustered using Perseus (Fig. 3). There are no specific traits referring to the new substrate, which is lupine moromi, as the only metabolic clusters all isolates from lupine moromi have in common are the ones for D-ribose, D-galactose and D-mannitol. The cluster for the metabolism of D-ribose and D-galactose appears to be conserved in the species *T. halophilus*, all other metabolic clusters are strain-dependent features. However, a higher occurrence of L-arabinose cluster *araBDAER* can be seen in isolates from lineage II (Fig. 3).

We found that for all strains isolated from lupine moromi and DSM strains the genomic prediction of functional metabolic cluster were correct besides for the metabolism of gluconate as no one the strains tested in the API test showed production of acid from gluconate (Table 2).

**Table 2:** Growth tests were performed with media supplied with 5 % NaCl (w/v) in either media. Determination of the optimal NaCl was done in MRS pH 5.7 after 48 h of incubation. Results of the API-CHL 50 test in API-CHL medium supplied with 2 % NaCl. All strains were able to produce acid from D-glucose, D-fructose, D-mannose, N-acetyl-Glucosamine, arbutin, Esculin/iron citrate, salicin, D-cellobiose, D-maltose, sucrose, D-trehalose, D-gentobiose, D-turanose. None of the strains could produce acid from erythritol, L-xylose, D-adonitol, methyl-beta-D-xylopyranosid, L-sorbose, L-rhamnose, dulcitol, inositol, inulin, adonitol, glycogen, xylitol, D-lyxose, D-fucose, L-fucose, L-arabitol, potassium gluconate, potassium-2-ketogluconate, potassium-5-ketogluconate; +, positive reaction; w, weak reaction; -, negative reaction; ND, not determined

				***T. halophilus***						
	***T. osmophilus***	subsp. ***flandriensis***	subsp. ***halophilus***							
	DSM 23765^**T**^	DSM 23766^**T**^	DSM 20339^**T**^	DSM 20337	TMW 2.2254	TMW 2.2256	TMW 2.2257	TMW 2.2263	TMW 2.2264	TMW 2.2266
Growth at/in:										
30°C in TSA	**+**	**+**	**+**	**+**	**+**	**+**	**+**	**+**	**+**	**+**
30°C in MRS	-	-	**+**	**+**	**+**	**+**	**+**	**+**	**+**	**+**
Optimal NaCl (w/v)	ND	ND	9%	8%	9%	10%	12%	10%	10%	9%
Fermentation of carbohydrates:										
Glycerol	-	-	-	-	-	**+**	**+**	-	-	-
D-Arabinose	-	**+**	-	-	-	-	-	-	-	-
L-Arabinose	-	-	**+**	**+**	-	**+**	-	**+**	-	**+**
D-Ribose	-	**+**	**+**	**+**	**+**	**+**	**+**	**+**	**+**	**+**
D-Xylose	-	-	-	-	-	-	-	-	-	**+**
D-Galactose	-	**w**	**+**	**+**	**+**	**+**	**+**	**+**	**+**	**+**
D-Mannitol	**+**	-	-	**+**	**+**	**+**	**+**	**+**	**+**	**+**
D-Sorbitol	-	**+**	-	**+**	-	**+**	**+**	**+**	-	-
Methyl-alpha-D-Mannopyranosid	**+**	-	-	-	-	-	**+**	-	-	-
Methyl-alpha-D-Glucopyranosid	**+**	**+**	**+**	**+**	**+**	**+**	**+**	-	-	-
Amygdalin	-	**+**	**+**	**+**	**+**	**+**	**+**	**+**	**+**	**+**
D-Lactose	-	-	-	-	-	**+**	-	**w**	-	-
D-Melibiose	-	**+**	-	**+**	**+**	-	-	-	**+**	-
D-Melecitose	-	-	**+**	-	-	-	**+**	-	-	-
D-Raffinose	-	**+**	-	-	-	-	-	-	-	-
D-Tagatose	-	-	**+**	**+**	**+**	**+**	**+**	**+**	-	**+**
D-Arabitol	**+**	-	**+**	-	**+**	**+**	**+**	**+**	**+**	-

### Amino acid metabolism and osmotolerance mechanisms

Variations in several amino acid degradation pathways contributing towards pH homeostasis and osmotolerance are present in the strains isolated from lupine moromi as well as in the rest of the set used. The major degradation pathway for arginine via the arginine deiminase (ADI) pathway encoded by *argRRABC* and *arcCRD* is strain dependent. Some of the strains have a disrupted operon or are missing several genes of the ADI pathway leading towards an inactive pathway [30]. Among the strains isolated from lupine moromi the strains TMW 2.2254, TMW 2.2263 and TMW 2.2266 have an incomplete ADI pathway due to absence of the second *argR* regulator, the genes *argB* and *argC* and the transporter with respective regulator *arcCRD*. Similar incomplete ADI pathways can be found in the strains DSM 23766^T^, D10, D-86, WJ7, NISL 7118 and 8C7. Indicating that a different pathway might compensate the loss of the ADI pathway.

The ability to decarboxylate aspartate *via* the aspartate decarboxylase (*aspD*, EC:4.1.1.12) is only present in the strains D10, D-86, NBRC 12172, NISL 7126, YA163, YG2, DSM 20339^T^, KUD23, TMW 2.2254 and TMW 2.2264. In the strain YA5 this decarboxylase seems to be frameshifted due to premature stop codon. The aspartate/alanine antiporter (*aspT*) is adjacent to the decarboxylase in all of these strains.

The ornithine cyclodeaminase (*odc*, EC:4.3.1.12) is present in all strains but in strain FBL3. In DSM 20337, D10 and D-86 the orf is splitted due to the end of a contig. The orf in the type strain DSM 20339^T^ is disrupted due to insertion of a transposase and is probably not functional.

The import of compatibles solutes in *T. halophilus* is mediated by the *opuA*, *opuC*, *busAB*, *opuABC* systems and the bcct transporter *betT*. The *busAB* and *opuC* systems are present in all strains. The orfs for the *opuAA* subunit of *opuABC* transporter in strain 8C7 are frameshifted due to a premature stop codon. The strain TMW 2.2266 did not harbor the complete *opuA* system putatively transporting glycine betaine and proline. The betaine-aldehyde dehydrogenase (*gbsA*, EC: 1.2.1.8), the choline dehydrogenase (*gbsB*, EC:1.1.1.1) and the HTH-type regulator *gbsR* are present in all strains but TMW 2.2266.

Genome analysis of the entire set for histidine (BAG14318.1, BAG14319.1) and tyrosine (WP_031944088.1) decarboxylases reveals that only *T. halophilus* subsp. *halophilus* 11 encodes for a histidine decarboxylase. None of the TMW strains produces any biogenic amines from histidine or tyrosine. Furthermore, using multiple annotation pipelines (NCBI, RAST, TIGR) a cluster consisting of four genes related to the agmatine deiminase pathway (agDI) was found in the strains D10, D-86, WJ7, KUD23 and TMW 2.2266. The cluster encodes an agmatine deiminase, a putrescine carbamoyl transferase, a LacI regulator and an amino acid permease.

### Bacteriophages and CRISPR/Cas systems in *Tetragenococcus halophilus*

Using Phaster “intact” bacteriophages in the genomes of DSM 23766^T^, KG12, NISL 7126, NBRC 12172, YA163, FBL3, TMW 2.2254, TMW 2.2256, TMW 2.2257, TMW 2.2263, TMW 2.2264 and TMW 2.2266 were identified. The program also correctly predicted the phage of the strain D-86 [31]. However, the phage of D10 was only predicted as questionable with a score of 80. A score of 90 would be considered as intact, this can be due to the sequence quality as the phage was shown to be lytic [32].

CRISPR/Cas systems are part of the defense against bacteriophages and foreign DNA. Nine different Cas type combinations with an evidence level of 4 were identified in 12 strains of *T. halophilus.* Two strain had an evidence level of 1. In 11 strains no marker proteins of a specific Cas type or with an evidence level of 0 were identified. Manual screening of Cas marker genes revealed that only in strains with an evidence level of 4, Cas proteins are present. The type of Cas systems does not correlate with any isolation source nor with the absence or presence of bacteriophages (Table S1).

### Bacteriocin production

Bacteriocins can positively contribute to the competitiveness of a strain within a fermentation broth. To identify potential bacteriocin producing strains BAGEL4 was used [33]. BAGEL did not find any orfs related to a Bacteriocins. BAGEL only detected one orf annotated as peptidoglycan DD-metalloendopeptidase in the strains DSM 20339^T^, 11, 8C7, NISL 7118, D-86, YA5, WJ7, YA163 and DSM 20337 has a 42 – 45 % (AA) similarity to a Zoocin A, a peptidoglycan hydrolase produced by *Streptococcus equi.*

## Discussion

In this study we characterized and compared *T. halophilus* strain isolated from lupine moromi with strains from different environment, in order to identify traits that might be necessary or wanted in a starter culture for the lupine moromi fermentation. Therefore, we compared these strains in terms of their salt tolerance, formation of biogenic amines, carbohydrate metabolism, potential bacteriocin production and the identified Cas systems.

**Fig. 2:**
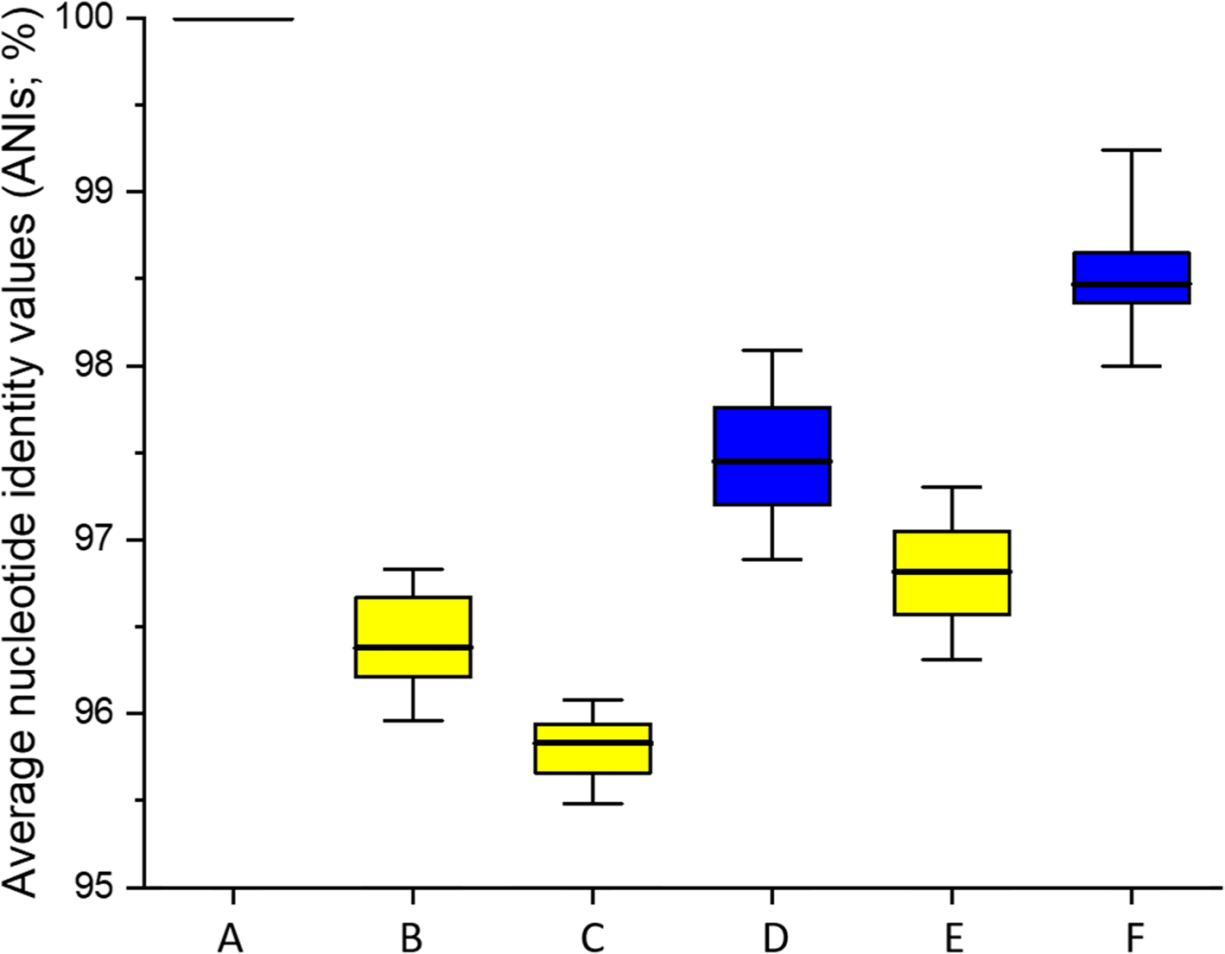
Pairwise average nucleotide identity values (ANI;%) of genome sequence belonging to the same lineage of *T. halophilus*. ANI values were calculated for the strain within the same linage or between lineages. A: Intralineage values of *T. halophilus* subsp. *flandriensis*, B: Interlineage values of *T. halophilus* subsp. *flandriensis* to the strains of Lineage I, C: Interlineage values of *T. halophilus* subsp. *flandriensis* to the strains of Lineage II, D: Intralineage values of *T. halophilus* lineage I, E: Interlineage values of *T. halophilus* lineage I to lineage II, F: Intralineage values of *T. halophilus *lineage II

Strains isolated from lupine moromi were compared to a representative set of publicly available genomes sequences from *T. halophilus* (Table 1) (Fig. S1*)*. The 16 S rRNA gene similarity ≥ 98.7 % with both subspecies type strains, DSM 20339^T^ and DSM 23766^T^, shows that all of the new isolates belong of the species *T. halophilus* (Fig. S2). Using concatenated housekeeping genes (*fusA, gyrA, gyrB, lepA, pyrG, recA, rpoD*), two lineages in the species *T. halophilus* could be revealed (Fig. 1, Fig. S3). All isolates from lupine moromi are all belong to the same lineage (lineage I). However, none of the type strains belong to this lineage. The separation into two lineages is further supported by the ANIb values (Fig. 2). Based on phenotypic (D-arabinose, D-lactose, D-raffinose, glycerol) and genotypic differences observed, as well as the different origins of the strains and the industrial relevance of thick juice degradation, two subspecies *T. halophilus* subsp. *halophilus* and *T. halophilus* subsp. *flandriensis* have been described previously [5]. Genomic analysis showed that some of the set criteria (e.g. D-lactose) are just strain dependent features, that in fact do not correlate with the subspecies affiliations. The isolates from lupine moromi cannot be clearly assigned towards one of the existing subspecies, based on the requirements for subspecies delineation. Furthermore, a potential third subspecies might be present as the *in silico* DDH values of the strains from lupine moromi range from 73.1 % to 77.1 % to the type strain of DSM 20339^T^ and 74.5 % - 77 % to the type strain DSM 23766^T^, as *in silico* DDH ≥ 79 % will be classified as same sub-species [34, 35]. These results indicate the possibility of a third subspecies and also demonstrate that the biodiversity in the species *T. halophilus* is greater than expected.

The core genome of the species *T. halophilus* consists of 1200 CDS of the Pan genome in size, this indicates that the species has a high biodiversity. A similar diversity can also be found in other LAB e.g. in *Limosilactobacillus reuteri* and *Lactobacillus delbrueckii*, both species with a demonstrated high species diversity and adaptation to different niches [36]. The carbohydrate utilization of several plant-based carbohydrates is conserved in *T. halophilus* (D-glucose, D-mannose, D-galactose, D-ribose, D-maltose). However, some of the pathways associated with salt tolerance and response salt stress (ADI, AgDI, aspDT) are strain dependent features among all members of the species *T. halophilus.*

**Fig. 3:**
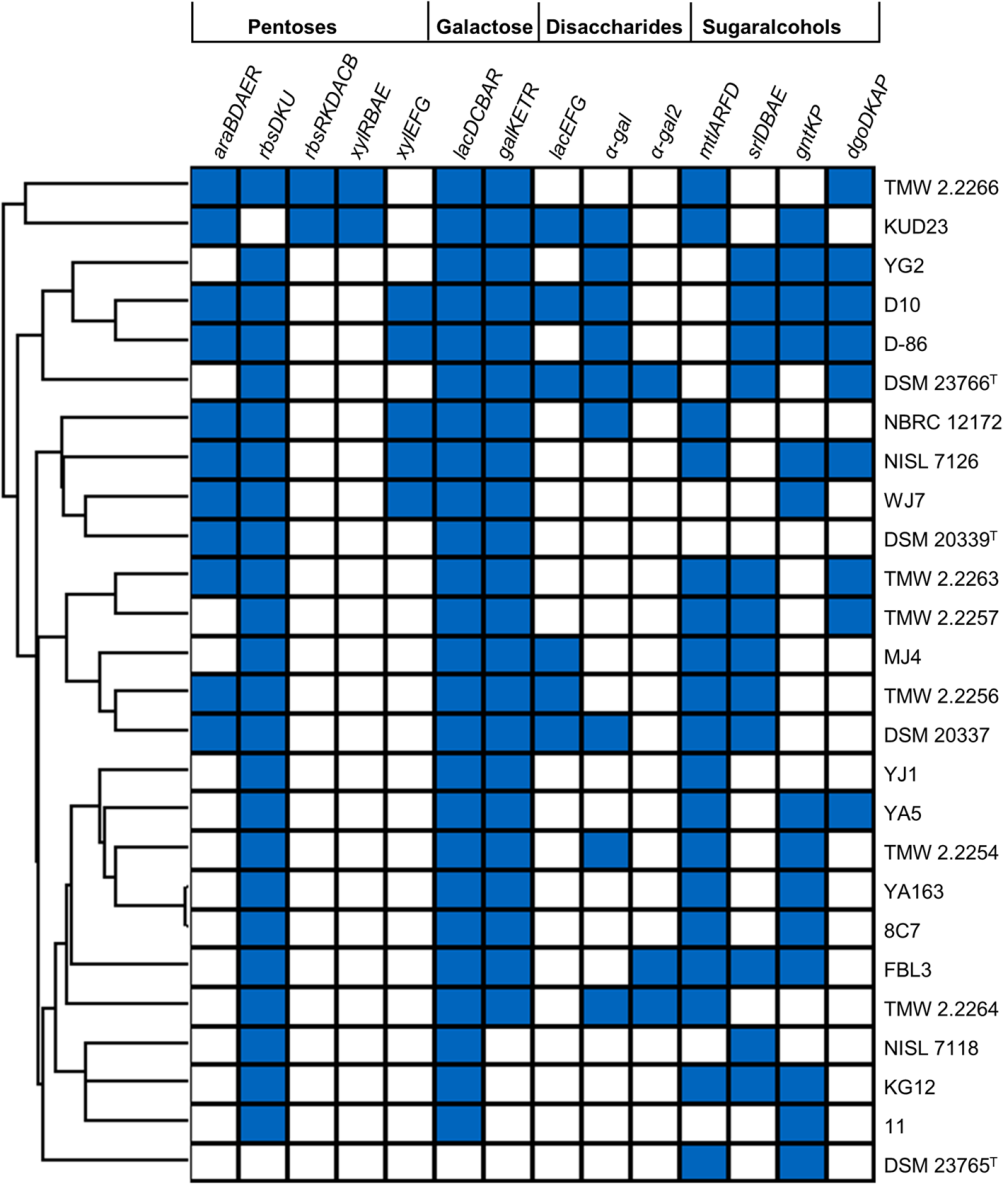
Hierarchical clustering of the functional carbohydrate metabolism cluster in *T. halophilus* using the hierarchical clustering function with Euclidean distance clustering in Perseus. Black box= functional and complete cluster. White box= incomplete/unfunctional or missing cluster. The strain *T. osmophilus* DSM 23765^T^ was used as an outgroup

Although soybeans and lupine seeds have comparable nutrient contents, the major difference is in the composition of lupine and soybean galactans. Galactans from soybeans have a higher L-arabinose content than the galactans from lupine seeds [37]. This may be the reason why the distribution of the *araBADER* operon is higher among strains from soybean fermentations (Fig. 3). Interestingly the distribution of clusters for the utilization of sugar alcohols (D-mannitol, D-sorbitol, gluconate, galactonate) is also the highest among isolates from lupine moromi. Although all the genes for gluconate metabolism are present in the strains- from lupine moromi, none of the strains produced acid from gluconate in the API 50 CHL test (Table 2). Considering the mechanisms towards pH homeostasis and amino acid degradation the isolates from lupine moromi do not show a clear adaptation towards a different environment as only strain dependent differences could be found.

To assess the starter culture suitability of the strains from lupine moromi, salt tolerance and formation of biogenic amines were analyzed. None of strains produced any tyramine or histamine in MRS supplied with the respective precursor amino acid. The absence of respective genes encoding decarboxylases was also confirmed by genomic analysis.

As citrulline, an intermediate of the ADI pathway, is associated with the formation of ethyl carbamate during the soy sauce fermentation [38], starter cultures should ideally be ADI negative to avoid the formation and accumulation of citrulline. Three strains from lupine moromi (TMW 2.2254, TMW 2.2263, TMW 2.2266) are ADI negative and could therefore be considered as potential starter strains. However, the strain TMW 2.2266 is AgDI positive and therefore is potentially not safe in terms in biogenic amines formation. Interestingly, strains only posses either a complete ADI or AgDI pathway but not both. Furthermore, the genomic analysis for potential prophages revealed that all of the isolates from lupine moromi had at least one predicted intact prophage. To avoid phage induced lysis of the starter strain and therefore the delay or stop of the directed fermentation as seen in other fermentations [39], a mixture of strains should be considered as starter culture. We could furthermore detect multiple different Cas types in the strains isolated from lupine moromi as well as in other strains from *T. halophilus*. Notably not all *T. halophilus* strains do possess a CRISPR/Cas system with no correlation to their isolation source (Table S1). However, as these are strain dependent differences, these could be used for strain identification and typing to track strains during a fermentation, as a similar approach was used in *Fructilactobacillus sanfranciscensis* [40]. Screening for bacteriocin production cluster of all the lupine moromi isolated strains could not detect any candidate genes. Which suits the strains well for a multi-strain culture and indicates that bacteriocins are not necessary for the domination of microbiota in lupine moromi.

## Conclusions

The phenotypical discrimination between strains from lupine moromi and other isolation sources is still presumably done at the carbohydrate level. The new environment, lupine moromi, selects only for strains from lineage I. Therefore, we proposed only strains from this lineage as potential starters for the lupine moromi fermentation. We could find that some traits and mechanisms associated with salt tolerance (*opuA, gbsAB*, ADI) are only strain dependent features. Phylogenetic analysis revealed three lineages within the species of *T. halophilus*. This delineation within the species is supported by DDH values <79 % for most of the strains in lineage I. Despite a given genomic coherency and distinguishability, at the time of writing we do not have sufficient data to provide a strong proposal for a third subspecies including strains of lineage I.

## Methods

### Isolation of strains and origin of type strains

Six new *T. halophilus* strains were isolated from the moromi fermentation of a novel lupine seed fermentation as described by Lülf et al., [41]. Toasted lupine seeds were soaked in water, cracked, mashed and subsequent fermented at 28 - 35 °C for two days with *Aspergillus oryzae* in an industrial fermentation of the Purvegan factory (Ramsen, Germany). The seeds taken from koji were mixed in the ratio 1:1.5 with tap water containing varying concentrations of NaCl (10 % – 20 % (w/v)) to establish different moromi fermentations [41]. Bacteria were isolated performing a serial dilution of moromi samples with Ringer solution (Merck) and were then streaked out on MRS agar plates with 5 % (w/v) NaCl and pH adjusted to 5.7 [42]. MRS plates were incubated at 30 °C for four days in an anaerobic jar with an AnaeroGen™ sachet (Thermo Scientific, Waltham, MA, USA) to create microaerobic conditions. The strain TMW 2.2254 was isolated from a fermentation with 10 % (w/v) NaCl after eight weeks. TMW 2.2257 was isolated from 13.5 % (w/v) NaCl after 12 weeks and the strain TMW 2.2263 was isolated from 15 % (w/v) NaCl after 12 weeks. Additionally, a second set of fermentations were inoculated with mature moromi from the Purvegan factory, also containing 10 % - 20 % (w/v) NaCl. From this second set, the strain TMW 2.2256 was isolated from a fermentation with 10 % (w/v) NaCl after two weeks. TMW 2.2264 was isolated from the inoculated moromi fermentation containing 15 % (w/v) NaCl after two weeks. TMW 2.2266 was isolated from the inoculated moromi containing 15 % (w/v) NaCl after 12 weeks. All isolates were identified *via* Matrix Assisted Laser Desorption Ionization - Time of Flight (MALDI-ToF) mass spectrometry (Bruker, Billerica, Massachusetts, USA). *T. halophilus* DSM 20337, *T. osmophilus* DSM 23765^T^, *T. halophilus* subsp. *flandriensis* DSM 23766^T^ and *T. halophilus* subsp. *halophilus* DSM 20339^T^ were from DSMZ (Braunschweig, Germany).

### Cultivation conditions

All strains were grown at 30 °C in a 15 ml or 50 ml closed tubes without shaking in MRS pH 5.7 [42] or TSA (Caso Bouillon) (Carl Roth, Karlsruhe, Germany) containing 5 % NaCl (w/v) (Carl Roth, Karlsruhe, Germany).

### DNA isolation and Genome sequencing

Genomic DNA was isolated from bacterial cultures grown in 10 ml MRS containing 5 % (w/v) NaCl using the E.Z.N.A Bacterial DNA-Kit (Omega bio-tek, Norcross, Georgia, USA) according to the manufacturer’s instructions. Isolated genomic DNA was sequenced by Eurofins Genomics (Konstanz, Germany) with Illumina HiSeq with the sequence mode NovaSeq 6000 S2 PE150 XP.

### Genome analyses

Assembly of the reads was done with the unicycler tool version 0.4.8 at the galaxy website (https://usegalaxy.eu/) with exclusion of contigs shorter of one kb and all other settings set to standard parameters. The assembled genomes were annotated using the NCBI PGAP, rapid annotations using subsystems technology (RAST) [43] and with an inhouse pipeline using the “The institute for Genomic Research” (TIGR) annotation. The average nucleotide indices (ANIb) were calculated with JSpeciesWS version 3.7.8 [44]. The **B**l**a**st **D**iagnostic **Ge**ne Finder (BADGE) was used to find diagnostic marker genes (DMGs) or unique CDS of the *T. halophilus* lineages and strains [45]. The predicted proteins of metabolic pathways were checked with pBlast (https://blast.ncbi.nlm.nih.gov/Blast.cgi) and smartBlast (https://blast.ncbi.nlm.nih.gov/smartblast/) from NCBI. Furthermore, the CDS search of NCBI (https://www.ncbi.nlm.nih.gov/Structure/cdd/wrpsb.cgi) was used to check for conserved domains in predicted proteins. Prophages sequences in the genome were predicted with the **PHA**ge **S**earch **T**ool **E**nhanced **R**elease (PHASTER) [46]. To check for bacteriocin production cluster the genomes were analyzed with BAGEL4 [33]. CRISPR loci and CAS proteins were identified using Crisprdb (https://crisprcas.i2bc.paris-saclay.fr). The Genome-to-Genome distance calculator ver. 2.1 was used to calculate the relatedness between different strains [47]. Hierarchical clustering of the functional clusters/genes was done with Perseus 1.6.14.0 using the hierarchical clustering function with the distance calculation set to Euclidean distance and the linkage set to average. The maximal number of iterations was set to 1.000 and all clusters/genes pictured in Fig. 3 were used for the distance calculations. All sequence alignments in this study were done in mega7 [48]. Sequences were aligned using Clustalω as implemented in mega7. Dendrograms were reconstructed using the neighbor-joining [49] or maximum-likelihood algorithm [50]. To construct the pan/core genome plot (Figure. S1) based on the amino acid sequences of the strains, CMG biotools 2.2 was used.

### Utilization of carbohydrates

The API 50 CHL (BioMérieux, Marcy l’Etoile, France) was used to identify different fermentation patterns of the isolates. To inoculate a test strip, an overnight culture was grown in MRS or TSA containing 5 % (w/v) NaCl. The OD_600nm_ of this culture was set to 0.3, washed with full strength Ringer’s solution (Merck, Darmstadt, Germany) and resuspended in API 50 CHL medium containing 2 % (w/v) NaCl. The test strips were evaluated after an incubation period of 48 h at 30 °C (Table 2).

### Formation of biogenic amines

To screen for production of biogenic amines (histamine and tyramine) in the newly isolated *T. halophilus* strains, the strains were cultivated in a modified MRS medium supplied with 0.005 % (w/v) pyridoxal 5-phosphate and 10 mM of LHistidine monochloride or L-Tyrosine di-sodium salt [51]. Cultivation was done in 1 ml medium in 1.5 ml Eppendorf tubes, which were inoculated with a single colony of each strain. The *Latilactobacillus sakei* TMW 1.1474 and *L. curvatus* TMW 1.595 were taken as positive control for histamine or tyramine production. The tubes were optically evaluated for a change in color after an incubation time of 48 h at 30 °C (Table S3).

### Determination of salt tolerance

Growth of each strain was recorded in MRS pH 5.7 supplied with different NaCl concentrations, to determine the strain specific optimal NaCl concentration. Therefore, 50 ml MRS with 2 % - 18 % (w/v) NaCl were inoculated with 500 µl of an overnight culture adjusted to an OD_600nm_ of 0.2 and resuspended in full strength Ringer’s solution (Merck, Darmstadt, Germany) and incubated 48 h at 30 °C without shaking. The OD_600nm_ was measured after 48 h in a SPECTROstar^nano^ plate reader (BMG, Labtech, Ortenberg, Germany) using a 96 well plate (Sarstedt, Nümbrecht, Germany) (Table 2).

### Accession numbers

The sequenced genomes are available on NCBI with the accession numbers: JACABZ000000000, JACACA000000000, JACACB000000000, JACACH000000000, JACACI000000000, JACACK000000000 and JACTUX000000000.

## Supplementary Information

**Additional file 1: Figure S1. ** Plotted pan- (blue) and core-(red) genome of *T.halophilus* strain. For every strain added thepan genome increases and the core genome decreases slightly after 17 strains added. Therefore, the core genome is considered as closed after the addition of every strain from this set. 1: DSM 20339^T^, 2: DSM 23766^T^, 3: NBRC 12172, 4: NISL 7126, 5: 11, 6: D10, 7: D-86, 8: KG12, 9: YA163, 10:YA5, 11: YG2, 12: WJ7, 13: DSM 20337, 14: NISL 7118, 15: KUD23, 16: MJ4, 17:FBL3, 18: YJ1, 19: TMW 2.2254, 20: TMW 2.2256, 21: TMW 2.2257, 22: TMW 2.2263, 23: TMW 2.2264, 24: TMW 2.2266, 25: 8C7.

**Additional file 2: Figure S2. ** A phylogenetic treeof the 16S rRNA gene sequences of the genus *Tetragenococcus* with the newlyisolated strains and the respective type strains of each species was constructed using the Neighbor-Joining method [49]. The accession number of the 16S rRNA of the type strains is given in brackets. For the newly isolated strains, the genome accession number is present inbrackets. The optimal tree with the sum of branch length = 0.09391473 is shown. Bootstrap values (1000 replicates) are shown next to the branches [52]. The tree is drawn to scale, with branchlengths in the same units as those of the evolutionary distances used to inferthe phylogenetic tree. The evolutionary distances were computed using theMaximum Composite Likelihood method [53]and are in the units of the number of base substitutions per site. Allpositions containing gaps and missing data were eliminated. There were a totalof 1214 positions in the final dataset. The type strain of *Enterococcus* *faecium* DSM 20477^T^ served as outgroup.

**Additional file 3: Figure S3. ** Phylogenetic tree of *T. halophilus* based on concatenated nucleotide sequences of the housekeeping genes(*fusA*, *gyrA*, *gyrB*, *lepA*, *pyrG*, *recA*, *rpoD*) using the Maximum Likelihood methodbased on the Tamura-Nei model [54]. Thetree with the highest log likelihood (-27635.30) is shown. The percentage oftrees in which the associated taxa clustered together is shown next to thebranches. Initial tree(s) for the heuristic search were obtained automaticallyby applying Neighbor-Join and BioNJ algorithms to a matrix of pairwisedistances estimated using the Maximum Composite Likelihood (MCL) approach, andthen selecting the topology with superior log likelihood value. The tree isdrawn to scale, with branch lengths measured in the number of substitutions persite. All positions containing gaps and missing data were eliminated. Therewere a total of 12057 positions in the final dataset. The strains isolated fromlupine moromi are marked in yellow. *T. osmophilus* DSM 23765^T^ wasused as an outgroup.

**Additional file 4: Table S1. ** CRISPR/Cas systems and intact prophages detectedin *T. halophilus*. The CRISPR/Cas sequences were identified using the CRISPRdb(https://crisprcas.i2bc.paris-saclay.fr). The evidence level indicates how manymarker proteins of a specific Cas type were found. Strains are ordered byisolation source, Cas type detected and evidence level. The intact prophageswere predicted using PHASTER (https://phaster.ca).

**Additional file 5: Table S2. **Comparative analysisusing BADGE [45] for the identification CDS specific to lineage I (Table S2 A)or lineage II (Table S2 B). BADGE could also identify some CDS only present insome strains of lineage II (Table S2 C). The Strains of each lineage are shown above and the presence or absence of a highly similar CDS is shown as a"+" or "-" in the respective column.

**Additional file 6: Table S3.** Formation of biogenic amines was tested as described by Bover-Cid et al. [51] in 1.5 ml Eppendorf tubes.Each strain was cultivated 48 h at 30 °C before optical evaluation. TMW 1.1474 and TMW 1.595 were used as positive control. ND, not detected.

## Data Availability

All genomes can be accessed at the NCBI website with the respective accession number given in Table 1. All data generated or analyzed during this study are included in this published article and its supplementary files.
