# Supplementary material for: The diversity among the species Tetragenococcus halophilus including new isolates from a lupine seed fermentation
Source: BMC Microbiol. 2021 Nov 20;21:320. doi: 10.1186/s12866-021-02381-1 (PMC8605565; doi:10.1186/s12866-021-02381-1)
Supplement: Supplementary file 1 — Additional file 1: Figure S1. Plotted pan- (blue) and core-(red) genome of T.halophilus strain. For every strain added thepan genome increases and the core genome decreases slightly after 17 strains added. Therefore, the core genome is considered as closed after the addition of every strain from this set. 1: DSM 20339T, 2: DSM 23766T, 3: NBRC 12172, 4: NISL 7126, 5: 11, 6: D10, 7: D-86, 8: KG12, 9: YA163, 10:YA5, 11: YG2, 12: WJ7, 13: DSM 20337, 14: NISL 7118, 15: KUD23, 16: MJ4, 17:FBL3, 18: YJ1, 19: TMW 2.2254, 20: TMW 2.2256, 21: TMW 2.2257, 22: TMW 2.2263, 23: TMW 2.2264, 24: TMW 2.2266, 25: 8C7. [file 12866_2021_2381_MOESM1_ESM.docx]

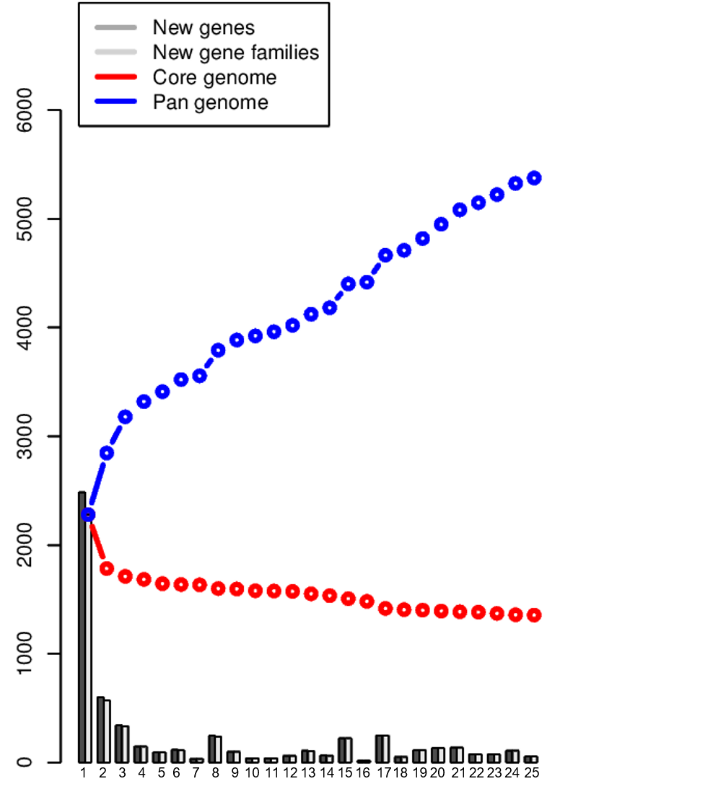


**Figure S1**: Plotted pan- (blue) and core-(red) genome of T. halophilus strain. For every strain added the pan genome increases and the core genome decreases slightly after 17 strains added. Therefore, the core genome is considered as closed after the addition of every strain from this set. 1: DSM 20339^T^, 2: DSM 23766^T^, 3: NBRC 12172, 4: NISL 7126, 5: 11, 6: D10, 7: D-86, 8: KG12, 9: YA163, 10: YA5, 11: YG2, 12: WJ7, 13: DSM 20337, 14: NISL 7118, 15: KUD23, 16: MJ4, 17: FBL3, 18: YJ1, 19: TMW 2.2254, 20: TMW 2.2256, 21: TMW 2.2257, 22: TMW 2.2263, 23: TMW 2.2264, 24: TMW 2.2266, 25: 8C7.
