# Supplementary material for: The diversity among the species Tetragenococcus halophilus including new isolates from a lupine seed fermentation
Source: BMC Microbiol. 2021 Nov 20;21:320. doi: 10.1186/s12866-021-02381-1 (PMC8605565; doi:10.1186/s12866-021-02381-1)
Supplement: Supplementary file 2 — Additional file 2: Figure S2. A phylogenetic treeof the 16S rRNA gene sequences of the genus Tetragenococcus with the newlyisolated strains and the respective type strains of each species was constructed using the Neighbor-Joining method [49]. The accession number of the 16S rRNA of the type strains is given in brackets. For the newly isolated strains, the genome accession number is present inbrackets. The optimal tree with the sum of branch length = 0.09391473 is shown. Bootstrap values (1000 replicates) are shown next to the branches [52]. The tree is drawn to scale, with branchlengths in the same units as those of the evolutionary distances used to inferthe phylogenetic tree. The evolutionary distances were computed using theMaximum Composite Likelihood method [53]and are in the units of the number of base substitutions per site. Allpositions containing gaps and missing data were eliminated. There were a totalof 1214 positions in the final dataset. The type strain of Enterococcus faecium DSM 20477T served as outgroup. [file 12866_2021_2381_MOESM2_ESM.docx]

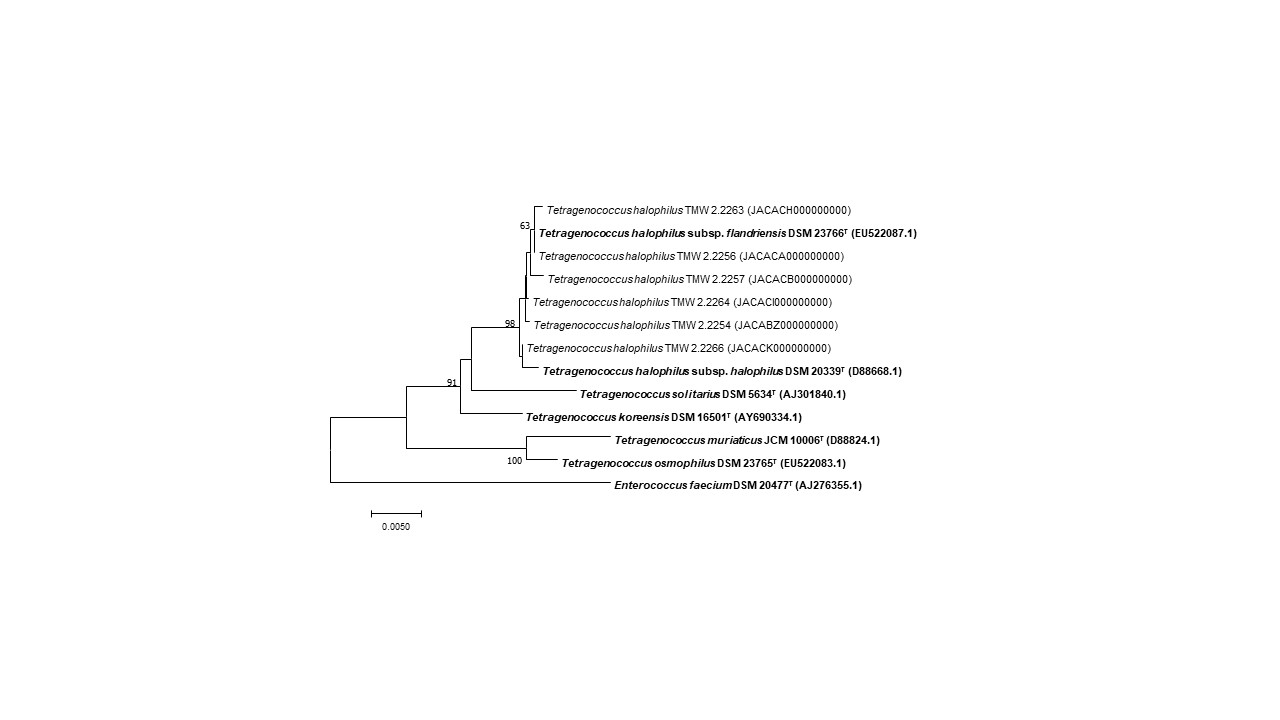


**Figure S2**: A phylogenetic tree of the 16S rRNA gene sequences of the genus Tetragenococcus with the newly isolated strains and the respective type strains of each species was constructed using the Neighbor-Joining method [49]. The accession number of the 16S rRNA of the type strains is given in brackets. For the newly isolated strains, the genome accession number is present in brackets. The optimal tree with the sum of branch length = 0.09391473 is shown. Bootstrap values (1000 replicates) are shown next to the branches [52]. The tree is drawn to scale, with branch lengths in the same units as those of the evolutionary distances used to infer the phylogenetic tree. The evolutionary distances were computed using the Maximum Composite Likelihood method [53] and are in the units of the number of base substitutions per site. All positions containing gaps and missing data were eliminated. There were a total of 1214 positions in the final dataset. The type strain of Enterococcus faecium DSM 20477^T^ served as outgroup.
