# Supplementary material for: The diversity among the species Tetragenococcus halophilus including new isolates from a lupine seed fermentation
Source: BMC Microbiol. 2021 Nov 20;21:320. doi: 10.1186/s12866-021-02381-1 (PMC8605565; doi:10.1186/s12866-021-02381-1)
Supplement: Supplementary file 3 — Additional file 3: Figure S3. Phylogenetic tree of T. halophilus based on concatenated nucleotide sequences of the housekeeping genes(fusA, gyrA, gyrB, lepA, pyrG, recA, rpoD) using the Maximum Likelihood methodbased on the Tamura-Nei model [54]. Thetree with the highest log likelihood (-27635.30) is shown. The percentage oftrees in which the associated taxa clustered together is shown next to thebranches. Initial tree(s) for the heuristic search were obtained automaticallyby applying Neighbor-Join and BioNJ algorithms to a matrix of pairwisedistances estimated using the Maximum Composite Likelihood (MCL) approach, andthen selecting the topology with superior log likelihood value. The tree isdrawn to scale, with branch lengths measured in the number of substitutions persite. All positions containing gaps and missing data were eliminated. Therewere a total of 12057 positions in the final dataset. The strains isolated fromlupine moromi are marked in yellow. T. osmophilus DSM 23765T wasused as an outgroup. [file 12866_2021_2381_MOESM3_ESM.docx]

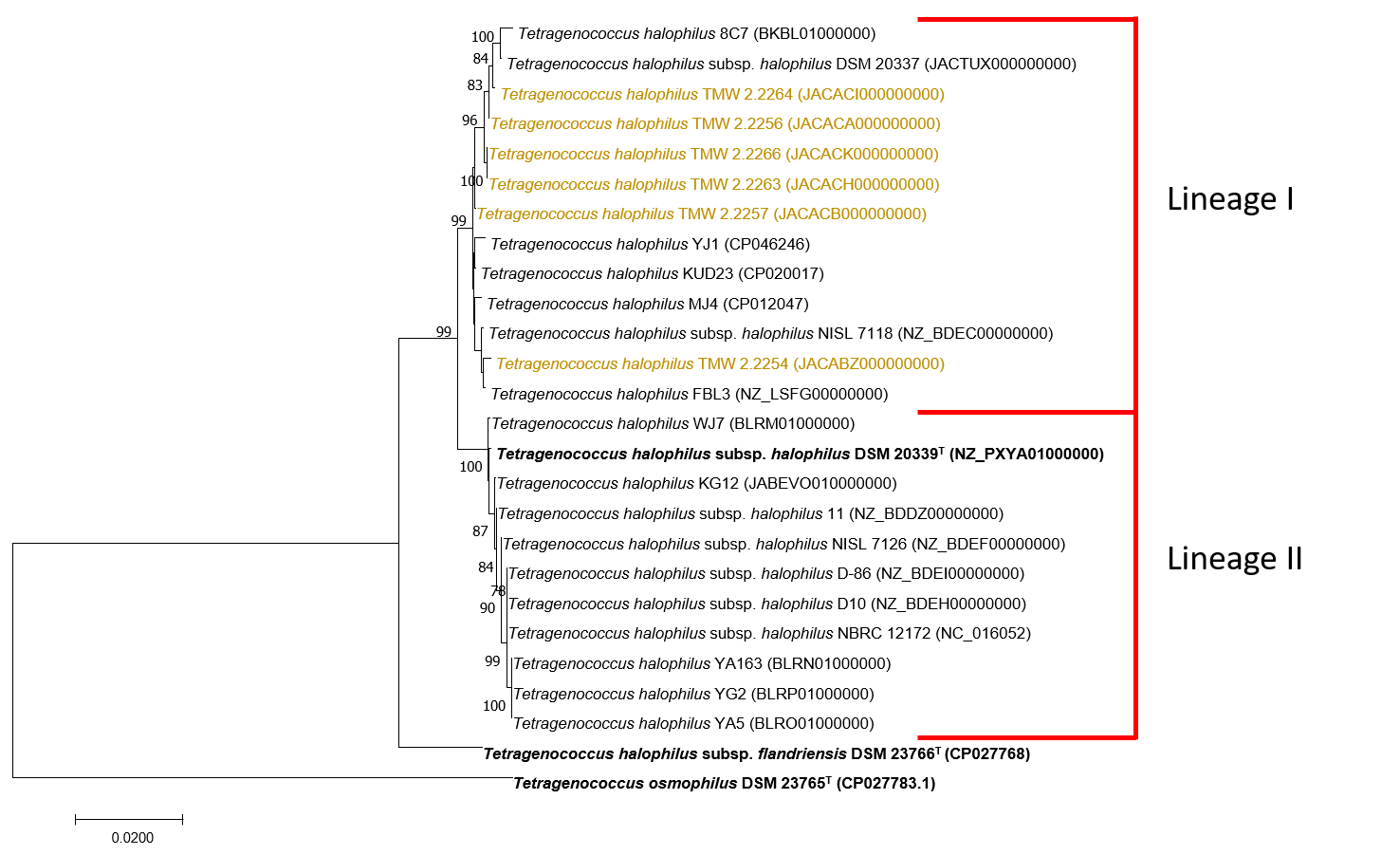


**Figure S3**: Phylogenetic tree of T. halophilus based on concatenated nucleotide sequences of the housekeeping genes (fusA, gyrA, gyrB, lepA, pyrG, recA, rpoD) using the Maximum Likelihood method based on the Tamura-Nei model [54]. The tree with the highest log likelihood (-27635.30) is shown. The percentage of trees in which the associated taxa clustered together is shown next to the branches. Initial tree(s) for the heuristic search were obtained automatically by applying Neighbor-Join and BioNJ algorithms to a matrix of pairwise distances estimated using the Maximum Composite Likelihood (MCL) approach, and then selecting the topology with superior log likelihood value. The tree is drawn to scale, with branch lengths measured in the number of substitutions per site. All positions containing gaps and missing data were eliminated. There were a total of 12057 positions in the final dataset. The strains isolated from lupine moromi are marked in yellow. T. osmophilus DSM 23765^T^ was used as an outgroup.
