# Supplementary material for: The diversity among the species Tetragenococcus halophilus including new isolates from a lupine seed fermentation
Source: BMC Microbiol. 2021 Nov 20;21:320. doi: 10.1186/s12866-021-02381-1 (PMC8605565; doi:10.1186/s12866-021-02381-1)
Supplement: Supplementary file 4 — Additional file 4: Table S1. CRISPR/Cas systems and intact prophages detectedin T. halophilus. The CRISPR/Cas sequences were identified using the CRISPRdb(https://crisprcas.i2bc.paris-saclay.fr). The evidence level indicates how manymarker proteins of a specific Cas type were found. Strains are ordered byisolation source, Cas type detected and evidence level. The intact prophageswere predicted using PHASTER (https://phaster.ca). [file 12866_2021_2381_MOESM4_ESM.docx]

**Table S1**: CRISPR/Cas systems and intact prophages detected in T. halophilus. The CRISPR/Cas sequences were identified using the CRISPRdb (https://crisprcas.i2bc.paris-saclay.fr). The evidence level indicates how many marker proteins of a specific Cas type were found. Strains are ordered by isolation source, Cas type detected and evidence level. The intact prophages were predicted using PHASTER (<https://phaster.ca>).

| Isolation source | Strain | CAS Type | Evidence level | Predicted intact prophages |
| --- | --- | --- | --- | --- |
| Lupine moromi | TMW 2.2257 | IIC + IIIAD | 4 | 3 |
| Lupine moromi | TMW 2.2264 | IC | 4 | 1 |
| Lupine moromi | TMW 2.2254 | IE | 4 | 1 |
| Lupine moromi | TMW 2.2256 | I | 1 | 1 |
| Lupine moromi | TMW 2.2266 | I | 1 | 2 |
| Lupine moromi | TMW 2.2263 | I | 0 | 2 |
| Soy sauce mash | 11 | IC + IIIC | 4 | 0 |
| Soy sauce mash | YG2 | IE + IC | 4 | 0 |
| Soy sauce mash | YA5 | IE + IC | 4 | 0 |
| Soy sauce mash | YA163 | IC | 4 | 1 |
| Korean Soy sauce | KG12 | IE | 4 | 2 |
| Soy sauce mash | NISL 7118 | I + IIIC | 0 | 0 |
| Soy sauce mash | DSM 20337 | I + IIIC | 0 | 0 |
| Soy sauce mash | D10 | I + IIIC | 0 | 0 |
| Soy sauce mash | D-86 | I + IIIC | 0 | 1 |
| Soy sauce mash | NISL 7126 | I + IIIC | 0 | 2 |
| Soy sauce mash | NBRC 12172 | X | 0 | 1 |
| Korean soypaste | KUD23 | IIIA | 4 | 0 |
| Fish sauce | YJ1 | IE + IIIAD | 4 | 0 |
| Anchovy fish sauce | MJ4 | IC + IIIA | 4 | 0 |
| Fish nukazuke | WJ7 | IIIC | 4 | 0 |
| Fish sauce | FBL3 | I + IIIC | 0 | 1 |
| Salted anchovy | DSM 20339^T^ | X | 0 | 0 |
| Degraded sugar beet juice | DSM 23766^T^ | X | 0 | 2 |
| Brie de meaux cheese rind | 8C7 | X | 0 | 0 |
