# Supplementary material for: The diversity among the species Tetragenococcus halophilus including new isolates from a lupine seed fermentation
Source: BMC Microbiol. 2021 Nov 20;21:320. doi: 10.1186/s12866-021-02381-1 (PMC8605565; doi:10.1186/s12866-021-02381-1)
Supplement: Supplementary file 6 — Additional file 6: Table S3. Formation of biogenic amines was tested as described by Bover-Cid et al. [51] in 1.5 ml Eppendorf tubes.Each strain was cultivated 48 h at 30 °C before optical evaluation. TMW 1.1474 and TMW 1.595 were used as positive control. ND, not detected. [file 12866_2021_2381_MOESM6_ESM.docx]

**Table S3**: Formation of biogenic amines was tested as described by Bover-Cid *et al*., [51] in 1.5 ml Eppendorf tubes. Each strain was cultivated 48 h at 30 °C before optical evaluation. TMW 1.1474 and TMW 1.595 were used as positive control. ND, not detected.

| Isolation source | Strain | Histamine | Tyramine |
| --- | --- | --- | --- |
| Lupine moromi | TMW 2.2254 | ND | ND |
| Lupine moromi | TMW 2.2256 | ND | ND |
| Lupine moromi | TMW 2.2257 | ND | ND |
| Lupine moromi | TMW 2.2263 | ND | ND |
| Lupine moromi | TMW 2.2264 | ND | ND |
| Lupine moromi | TMW 2.2266 | ND | ND |
| Soy sauce mash | DSM 20337 | ND | ND |
| Salted anchovy | DSM 20339^T^ | ND | ND |
| Degraded sugar beet juice | DSM 23766^T^ | ND | ND |
| Sauerkraut | TMW 1.1474 | positive | ND |
| Starter preparation | TMW 1.595 | ND | positive |
